# Presence of red flags in patients with cerebral venous sinus thrombosis admitted to the emergency department because of headache: A STROBE compliant cohort-study

**DOI:** 10.1097/MD.0000000000020900

**Published:** 2020-07-17

**Authors:** David García-Azorín, Mariana H.G. Monje, Nuria González-García, Ángel L. Guerrero, Jesús Porta-Etessam

**Affiliations:** a Headache Unit, Neurology Department. Hospital Universitario Clínico de Valladolid. Valladolid; b Headache Unit, Neurology Department. Hospital Universitario Clínico San Carlos, Madrid; c Institute for Biomedical Research of Salamanca, IBSAL, Salamanca, Spain.

**Keywords:** diagnosis, headache disorders, intracranial, secondary, sinus thrombosis, venous thrombosis

## Abstract

Cerebral venous sinus thrombosis (CVST) is a cause of secondary headache with substantial morbimortality. Headache dominates the clinical presentation, but no typical phenotype has been described. We aim to evaluate the presence of red flags in headache in patients with confirmed CVST at the moment of emergency department (ED) presentation.

Retrospective STROBE compliant cohort study including patients with confirmed CVST that consulted because of headache at the ED. We analyzed presence and type of red flags at the moment of consult. We evaluated whether CVST was suspected at the moment of imaging request and analyzed delay in the diagnosis.

Nineteen patients fulfilled inclusion and exclusion criteria. Mean age was 48.5 years, 47.4% were female. All the studied patients exhibited at least 1 red flag, being abnormal neurological examination the most frequent (79%), followed by the presence of other neurological symptoms (68%), alarm data related with headache phenotype (63%), or risk factors concerning prior medical history (47%). Temporal pattern of the headache was acute in 42.1%, thunderclap in 31.6%, and subacute in 26.3%. In none patient CVST was the specific suspicion when imaging was requested. Median time since headache onset and ED presentation was 84 hours, being different in patients with associated symptoms (48 hours) when compared with isolated headache patients (168 hours). Time since ED presentation and the diagnosis also differed between the 2 groups, being more prolonged in patients with an isolated headache at presentation.

Headache attributed with CVST did not exhibit any distinctive phenotype, but all the patients presented some red flag, being abnormal neurological examination the most frequent.

## 1 Introduction

Cerebral venous sinus thrombosis (CVST) is a rare cause of stroke.^[1]^ Its incidence is 1.32 cases per 100,000 patients/year but increases in middle-aged women, among which it might affect up to 2.78/100,000 patients/year.^[2]^ The epidemiological picture of CVST is a middle-aged woman, being 73.7% of patients female aged 39.1 years in mean.^[3,4]^ Its prognosis with no adequate treatment is gloomy, with a mortality rate around 7.7% to 8.3%.^[3,4]^ Given the availability and efficacy of anticoagulation, diagnosis should be done as soon as possible.^[4,5]^

Diagnostic delay is common, around 3 days between hospital admission and diagnosis,^[3,4]^ probably related with the clinical heterogeneity, the lack of awareness, and the need of specific radiological imaging sequences for its adequate diagnosis.^[3,4,6,7]^ Clinical presentation of CVST is variable, begin considered as one of the “great mimickers” of Neurology. Phenotype might get influenced by the topography of the affected sinus,^[6]^ the existence of intracranial hypertension, and the presence of complications such as subarachnoid hemorrhage of venous infarcts.^[6,7]^

Headache is, by far, the most frequent symptom at onset, present in 88.8% of patients.^[6–13]^ It is usually accompanied by other neurological symptoms, but in 22.7% to 45.0% of the patients represents the sole symptom.^[6,8–10]^ Headache phenotype is polymorph, sometimes even mimicking “benign” headaches. Tension-type headache is a common misdiagnosis of CVST, as pain can be continuous in 88%, throbbing in 76% and progressive in 64%^[12,13]^; nevertheless, the needed number prior episodes contradicts it. The International Classification of Headache Disorders, 3rd edition (ICHD-3) does not require any specific headache phenotype for the diagnosis.^[14]^ When headache is the only presenting symptom, the diagnostic delay might prolong up to 13.1 days.^[12,15,16]^

Secondary headaches (SH) are those produced by a cause able to originate headache.^[17]^ The temporal relation with the onset, aggravation or improvement is required in the causation assumption.^[14]^ Due to the lack of specific biomarkers for headache in the Emergency Department (ED), the detection of SH is still based on the presence of red flags. Presence of red flags in headache attributed to CVST have been partially described^[9,13]^; however, presence of identifiable red flags at the ED admission has not been analyzed yet.

In this study, we aim to evaluate if a SH can be suspected due to the presence of any red flag at the moment of ED consultation in a series of CVST patients. Due to the needed index of suspicion and specific imaging modalities, we also pretend to evaluate whether suspicion of CVST changes the study work-up and reduces the time to diagnosis.

## 2 Material and methods

We conducted an observational retrospective study. Our study population included patients with confirmed CVST that consulted at the ED because of headache. Our inclusion criteria were: CVST confirmed by magnetic resonance venography, cranial tomography venography or angiography; presenting at the ED; complaining because of headache at ED presentation; fulfilling ICHD-3 criteria for headache attributed to cerebral venous thrombosis.^[18]^ Exclusion criteria were: isolated cavernous sinus thrombosis; infective thrombophlebitis; isolated Cortical venous thrombosis; unclear diagnosis after radiological reevaluation from an experienced neuroradiologist.

The study took place in University Hospital Clinico San Carlos from Madrid, a third-level public hospital with a reference population of 1 million inhabitants. The study period was between January 2009 and May 2015. We followed retrospectively all the patients since the ED presentation until the confirmation of the diagnosis.

We screened all the available cases from the Hospital General Database, the ED specific database, and the Radiology Department database by ICD codes. Two different researchers reviewed the cases for eligibility and a third neurologist solved the disputes. We tried to avoid selection bias by doing a wide search and reviewing a high number of records.

### 2.1 Data extraction

We carefully reviewed clinical information from the ED charts, evaluating demographical information such as age and sex. Information was obtained from computerized medical records.

**Figure 1:**
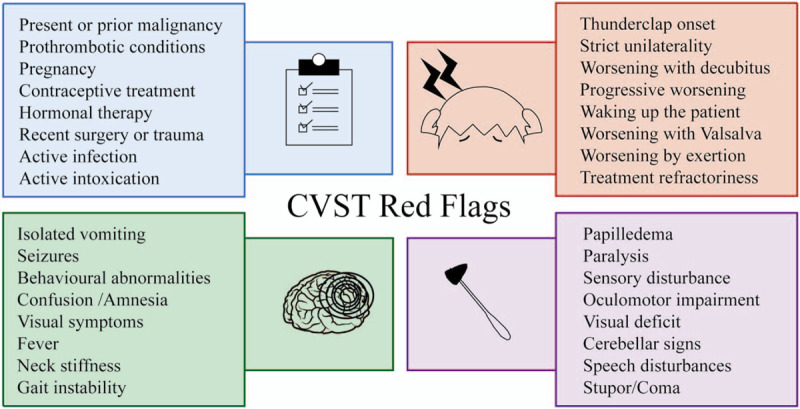
Red flags in cerebral venous sinus thrombosis. Classification of red flags in patients with cerebral venous thrombosis adapted considering previous authors publications.^[1,6,7,13,17,19]^

Two headache specialists evaluated the clinical data analyzing the presence of red flags at the moment of ED visit. We classified the clinical data into 4 categories depending on the possible red flag. We adapted the classification of red flags to the CVST setting considering previous authors publications^[1,6,7,13,17]^ (Fig. 1). The first category was related with medical history conditions that could be associated with CVST. The second category was the presence of red flags related to headache description and phenotype. The third group included red flags due to the existence of other neurological or systemic symptoms other than headache. We described nausea and vomiting presence by separate because they can be present both in primary headache and SH disorders. The last group was the presence of abnormal neurological signs detected in the neurological examination. In those red flags that could be either a symptom or a sign, for example, neuro-ophthalmological red flags, we considered symptoms if they were referred by patients and signs if they were observed during the examination. The components of each category are detailed in Figure 1

We classified patients based on the presence of only headache or existence of other symptoms. According previous studies,^[6,7,9,11]^ we defined *isolated headache* if at the moment of presentation, the patient did not complain about other neurological or systemic symptoms. If the patient reported other symptoms, it was defined as *headache plus* patient. In line with previous studies^[6,7,9,11]^ we differentiated 3 temporal patterns of headache depending on the time between the onset and the moment of maximal intensity: thunderclap, if the most severe intensity was reached within the first minute; acute, if it took <24 to reach the maximum intensity; and progressive, if it was after 24 hours.^[12]^

Concerning diagnosis, we evaluated if fundoscopy was done. We also analyzed the time between the onset of the symptoms, ED presentation, first radiological examination, and final diagnosis. We considered whether CVST was suspected as the secondary cause producing the headache at the moment of the cranial tomography (CT) petition. We reviewed which imaging examination was done after the first imaging depending the results.

Local ethics committee board approved the study (CP14/425-E). The study followed the Strengthening the Reporting of Observational in Epidemiology (STROBE) guidelines.^[19]^

### 2.2 Statistical analysis

The primary endpoint of the study was to evaluate the presence and type of any red flags at the moment of ED presentation. Secondary endpoints were to analyze: whether funduscopic examination was done in all cases; whether CVST was suspected at the moment of neuroimaging request, and whether delay in the consultation and diagnosis changed between the groups.

Qualitative variables are presented as frequency and percentage. Continuous variables are presented as mean and standard deviation (SD) in case of normal distribution or median and interquartile range (IQR) in case of non-normality. Normal distribution was evaluated with Kolmogorov-Smirnov test. In the case that some items were not detailed in the report, missing data were managed by doing Complete Case Analysis. We used *χ*^2^ test or Fisher exact test for the contrast of qualitative variables, Student *t* test when comparing qualitative and quantitative variables, or Mann-Whitney *U* test if distribution was not normal distribution and Pearson test in the comparison of quantitative variables, with Bonferroni correction in case of multiple comparisons. We considered an alpha level of 5%. Statistical analysis was performed with SPSS v20.0 (IBM Corp, Armonk, NY).

## 3 Results

During the study period, 31 patients were diagnosed of CSVT but 7 did not presented headache at onset. We excluded 3 patients because of isolated cavernous sinus thrombosis and 2 because of infective thrombophlebitis. Finally, 19 patients fulfilled both inclusion and exclusion criteria. Mean age of our sample was 48.5 years (SD: 20.7) and 47.4% of patients were female. Six patients complained only about headache and 13 described other symptoms as well (Table 1).

**Table 1:**
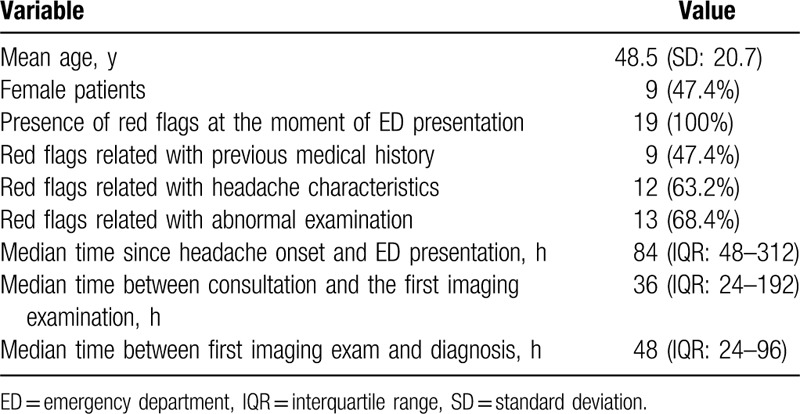
Summary of the demographic and clinical variables.

### 3.1 Headache characteristics

**Figure 2:**
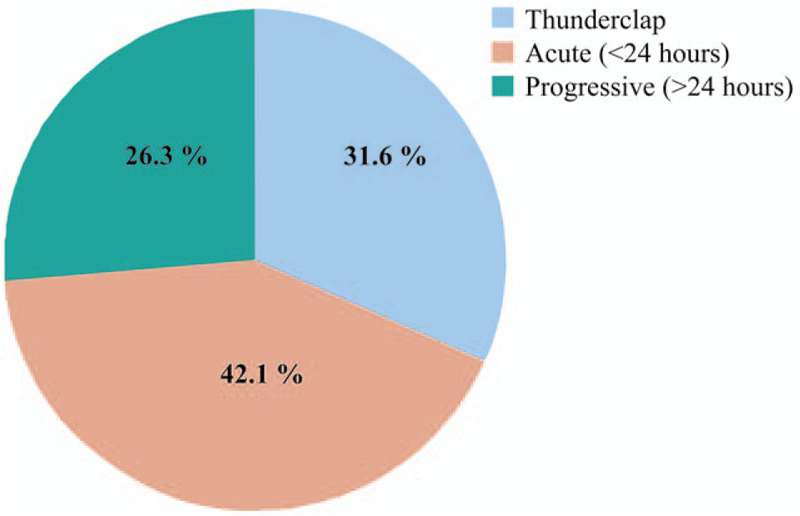
Temporal pattern of headache at presentation of patients with cerebral venous sinus thrombosis. Note how the high percentage of patients presented with thunderclap pattern of headache.

Headache temporal pattern is represented in the Figure 2. The most frequent pain pattern was pressing (63.2%). It was described as holocranial in 11 patients (57.9%), in 5 (26.3%) as hemicranial, in 2 as occipital, and in 1 single case frontal. Intensity was severe in 12 patients (63.2%) and moderate in 7 (36.8%). No patient referred headache intensity as mild.

### 3.2 Red flags presence

**Figure 3:**
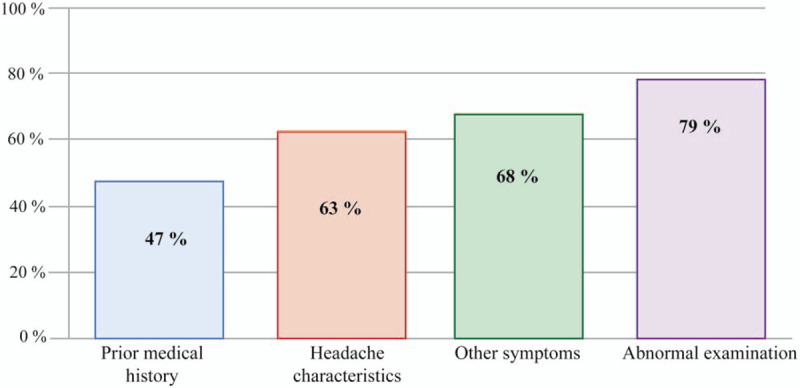
Reg Flags exhibited by patients with cerebral venous sinus thrombosis. All the patients have some red flags. Note how the presence of red flags in headache characteristics is present in more than half of the patients.

All the patients exhibited at least 1 red flag at the moment of ED presentation. Distribution of the different red flags within the 4 proposed categories is presented in Figure 3.

In 9 patients (47.4%) there were some recognizable alarm data about their previous medical conditions at presentation: 4 had previous active malignancy (21.1%), 4 reported previous venous thromboembolic events (21.1%), and 1 patient was under hormonal replacement treatment (5.3%).

Twelve patients (63.2%) described some alarm data related with headache characteristics. The most frequent was the progressive worsening of headache in 7 patients (36.8%), followed by thunderclap onset (6 patients, 31.6%), interruption of sleep (6, 31.6%), resistance to symptomatic treatment (5, 26.3%), worsening with decubitus (5, 26.3%), and aggravation with Valsalva maneuvers (4, 21.1%).

Thirteen patients (68.4%) referred other abnormal neurological symptoms. The most prevalent were neuro-ophthalmologic symptoms (7 cases, 36.8%), followed by motor weakness (5 cases, 26.3%), abnormal consciousness level (4 cases, 21.1%), behavioral disturbances (4 cases, 21.1%), fever (3 cases, 15.8%), seizures (2 cases, 10.5%), speech disturbances and gait instability in 1 patient each (5.3%). Nausea and vomiting was present in 8 patients (42.1%).

Neurological examination was abnormal in 15 patients (78.9%). The most frequently found signs were paresis (8 cases, 42.1%; 7 cases of hemiparesis, and 1 case of facial palsy), neuro-ophthalmologic signs (7 cases, 36.8%), sensory deficits (5 cases, 26.3%), and aphasia or decreased level of consciousness (2 patients each, 10.5%).

### 3.3 Funduscopic examination

Funduscopy was performed in only 12 patients (63.2%). It was more frequently done in patients with isolated headache (75%) than in those with headache plus other symptoms (54.5%). In 10 cases (83.3% of the examined) papilledema was present. All patients with isolated headache at presentation exhibited papilledema.

### 3.4 CVST index of suspicion

When the first imaging examination was ordered, a SH was suspected in all the cases. However, in none of the requests, “CVST,” “venous examination,” or “thrombosis” was mentioned. In the first CT, 11 cases presented some abnormal finding, but only 1 case was considered highly suggestive of CVST. In patients with a first abnormal CT scan, a second urgent neuroimaging with vascular evaluation was requested in 77.8% of cases, whereas in patients with a first normal CT scan, another urgent neuroimaging with vascular evaluation was done only in 27.2% of patients.

### 3.5 Delay in diagnosis

**Figure 4:**
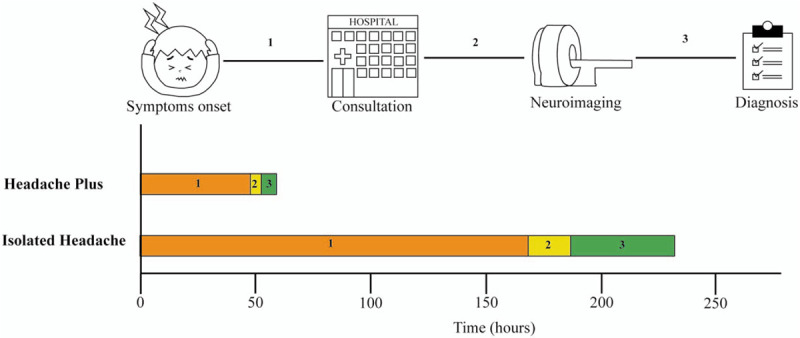
Graphical representation of the timing sequence since de clinical symptoms’ onset and the diagnosis of cerebral venous sinus thrombosis. Isolated headache clinical manifestation patients (yellow) has a more considerable time from symptoms onset to consultation (1) compared to a headache plus patients (red) (*P* < 0.05). The time between consultation and first neuroimaging (2) and the time between first neuroimaging and diagnosis (3) was shorter in headache plus patients, although it did not reach significant statistical difference (*P* = 0.1).

Median time since headache onset and ED presentation was 84 hours (IQR: 48–312). Patients with headache plus other symptoms at presentation consulted earlier than those with isolated headache (48 vs 168 hours, *P* < .05). Median time between consultation and the first imaging examination was 36 hours (IQR: 24–192) and between first examination and diagnosis 48 hours (IQR: 24–96). Time between ED consult and first imaging examination and time between first imaging examination and diagnosis tended to be shorter in patients with headache plus than patients with isolated headache, albeit differences were not statistically significant (*P* = .1 in both) (Fig. 4).

## 4 Discussion

In this study we analyzed whether CVST can be suspected in the ED because of the presence of red flags. We also analyzed which red flag group is more frequently abnormal and the impact of red flags presence in the diagnostic work-up and delay.

At the moment of ED presentation, all the patients presented at least 1 red flag and a SH was suspected in all cases. Despite its potential consequences, CVST was not specifically mentioned in any patient. However, we found that diagnosis was delayed in many cases and specific imaging modalities were requested seldom.

During the study period, 31 patients were diagnosed of CVST. After excluding those patients with infective thrombophlebitis or isolated cavernous sinus thrombosis, headache was the most frequent symptom of presentation in CVST patients in our sample, present at ED admission in 19 of 26 cases (73.0%). Many previous authors have highlighted the importance of headache as the key symptom in CVST diagnosis.^[6–13]^

In our sample, all the patients had red flags at the moment of ED presentation. The most frequent red flags were encoded in the group of abnormal signs in the neurological examination, present in 78.9% of patients. In 68.4% of patients, headache description contained some alarm characteristic and in 68.4% other alarm symptoms other than headache were present. Finally, some medical condition increasing the risk of a SH was identified at ED presentation in 47.4% of patients.

The clinical presentation may be related with CSVT extension. Venous sinus thrombosis is a dynamic process, so if venous drainage remains obstructed the intracranial hypertension is expected to rise. This may lead to a worsening of symptoms due to intracranial hypertension, venous infarcts, and even hemorrhagic infarcts or subarachnoid hemorrhage.^[8]^ The presence of hemorrhage has been also associated with seizures and the involvement of the deep venous system with decreased level of consciousness. Additionally, some authors described that the distension of the transverse sinus may be responsible of the lateralized pain.^[11,12]^

History and examination can give some clues about the underlying pathophysiological process.^[20,21]^ Symptoms such as worsening with decubitus, morning predominance, vomiting without previous nausea, or blurred vision, or signs such as papiledema or sixth nerve palsy can reflect intracranial hypertension. Seizures or focal signs might suggest venous infarcts or subarachnoid hemorrhage. The most useful manoeuvre in our sample was neurological examination, which should be always performed and might include fundus examination.

Neither us nor other authors have found any specific pattern of headache^[22]^; the main “chameleon” is tension-type headache. Nevertheless, in our sample at least two-thirds of patients described red flags about headache description, which would contradict TTH diagnosis. It is typical that there is not any distinctive phenotype, but in our sample, all patients exhibited some red flag, so primary headache disorders should be diagnosed only if no better explanation can be found.^[8,9,12]^

Among the possible medical history, attention should be focused on conditions that may predispose to suffer a CVST. Classically it has been classified depending on which part of the Virchow triad was altered^[23,24]^: hypercoagulability, hemodynamic changes, or endothelial injury. The most frequent ones are changes in the composition of the blood, such as acquired hypercoagulability states, mainly secondary to oral contraceptives, pregnancy, puerperium, but also inherited ones, as prothrombin mutation G20210A, Factor V Leyden, protein C and protein S deficiency. Second, the susceptibility to CVST can be related to endothelial damage: secondary to infections, inflammatory diseases, malignancies, mechanical causes, or trauma. Last but not least, hemodynamic changes with stasis of the blood might contribute to the problem, as it frequently happens in dehydration and secondarily in intracranial hypertension.^[23]^

Local causes of endothelial damage are less frequent in CVST than in other organ-specific thrombosis (34% compared with 73% to 88% as it is found in portal, renal, or pulmonary veins thrombosis), so systemic entities that may predispose to thrombosis should be considered.^[25]^ Some of these conditions can be identified in history. Attention should be paid, not only because it may support the diagnosis, but also because some of these conditions could precise a specific and prompt treatment, as in the case of Behçet disease.

In our sample, a secondary entity was suspected in all the patients when neuroimaging was ordered. Despite of that, diagnosis was deferred in many cases. It has previously described that diagnosis in patients with isolated headache may be delayed.^[12]^ Theoretically, if SH is suspected, even if basal CT is normal, a contrast-enhanced CT or MRI should be done. If CSVT diagnosis is a possibility, venous sequences should be included in the study.

In light with our findings, CVST should be considered also in the differential diagnosis of thunderclap headache. Venous-specific imaging sequences should be included because about one-third of CVST patients in our sample and previous studies had a sudden onset of headache.^[9]^

Potential limitations of our work are the: the small sample size; the retrospective nature of the study, which could underreport some symptoms that were not identified in the medical records and with higher interobserver variability; the participation of a single center; the risk of biases. Among the strengths of our study, it is the first study that specifically analyzed presence of red flags in CVST patients; we adapted the classification of red flags to this condition and we avoided false diagnosis by the specific review of all imaging sequences.

## 5 Conclusions

Headache is the most frequent presenting symptom in patients with CVST, but it is not universal. CVST-related headache typically has not a distinctive phenotype. All the patients of our sample presented some red flag in the medical history, clinical presentation, or neurological examination. Imaging of the cerebral venous system should be considered in patients with headache and red flags.

## Author contributions

DGA, MHGM, NGG collected the data; DGA, MHGM analyzed the data; NGG, AGP, JPE interpreted the data; DGA, MHGM wrote the draft; NGG, AGP, JPE reviewed it for intellectual content. All authors approved the final versions.
